# Resilience in maternal and child nutrition outcomes in a refugee-hosting community in Cameroon: A quasi-experimental study

**DOI:** 10.1016/j.heliyon.2022.e12096

**Published:** 2022-12-05

**Authors:** Lambed Tatah, Tharcisse Nkunzimana, Louise Foley, Alan de Brauw, Jose Manuel Rodriguez-Llanes

**Affiliations:** a MRC Epidemiology Unit, University of Cambridge, Cambridge, UK; b Delegation of the European Union to the Republic of Niger, Niamey, Niger; c International Food Policy Research Institute, Washington, USA; d European Commission Joint Research Centre, Ispra, Italy

**Keywords:** Resilience, Nutrition outcomes, Refugee-hosting community, Cameroon

## Abstract

Refugees may be perceived as a burden to their host communities, and nutrition insecurity is a critical area of contention. We explored the relationship between refugee presence and a host community’s resilience in nutrition outcomes in Cameroon. We also tested an analytical framework for evaluating community resilience during shocks. We used data from repeated cross-sectional Demographic and Health Surveys in Cameroon (2004 and 2011), data on refugee movement, and data on extreme climatic events, epidemics, and conflicts from multiple sources. Outcome variables were maternal underweight, maternal anaemia, and child underweight, anaemia, stunting and wasting. The exposure variable was residence within an area in which refugees settled. We used a genetic matching algorithm to select controls from the rest of the country after excluding areas experiencing concurrent shocks. We used a difference-in-differences analysis to compare outcomes between the exposed and control areas. The 2004 survey comprised 10,656 women and 8,125 children, while the 2011 survey comprised 15,426 women and 11,732 children. Apart from anaemia which showed a decreasing trend in both the refugee-hosting community and the rest of the country, all other indicators (wasting, underweight and stunting) showed increasing trends in the refugee-hosting community but decreasing trends in the rest of the country. The matched control group showed a similar trend of decreasing trend for all the indicators. Controlled comparisons showed no evidence of an association between changes in nutrition outcomes and the presence of refugees. These findings contest a common perception that refugees negatively impact hosting communities. The difference-in-differences analysis and an improved matching technique offer a method for exploring the resilience of communities to shocks.

## Highlights

• Host communities often blame refugees for their problems, but analyses of host resilience in nutrition security are limited.
• We approached this gap using a difference-in-differences analysis and multiple nutrition outcomes in women and children.
• We show that a refugee-hosting community can maintain stable nutrition outcomes over time, even if levels are suboptimal.
• These findings refute a common claim that refugees are an additional burden to hosting communities.
• Our findings demonstrate a way of conceptualising resilience in further studies for various exposures and outcomes.

## 1 Introduction

Emerging literature addresses the impacts of refugees on host communities, notably on the competition for limited resources, labour markets and trade [1]; environmental and infrastructural degradation [2]; disease propagation [3]; or on violence and crime [3]. These impacts may have direct and indirect consequences for nutrition security in host communities. Our understanding of such implications is still minimal, partly because of (i) the methodological challenges associated with the isolation of the effects of refugees on host communities and (ii) the diversity of approaches and outcome indicators needed to measure dietary, food and nutrition security [4].

The rising rate of climatic and conflict-related disasters is increasing the number of displaced persons. With over 30 million refugees today (and the number rising steadily in recent years) [5], how host communities cope with refugees is of utmost importance to researchers, policymakers and the communities concerned. As refugees flee persecution and conflict, often leaving behind their livelihoods, histories, aspirations and dreams to survive elsewhere at the mercy of well-wishers and mandated organisations, they attract more attention. Consequently, most studies tend to focus on the displaced [6]. The host communities, in most cases, while often fragile in many ways [6], are less studied. Any additional stress on resources in these fragile communities can create tension between the refugees and their hosts [7]. Ensuring resilience to nutrition insecurity in the host community is a significant step toward improving overall outcomes for both refugees and hosts and promoting the integration of refugees into communities.

Contrary to the current focus on the problems caused by mass displacement and migration, refugees can positively impact host communities. We showed in a previous study, in which we analysed the impact of refugees on the host’s primary healthcare system in Cameroon, that the presence of refugees had no detrimental effects on maternal and child primary healthcare indicators. For the most part, these indicators improved [8]. In other studies that examined economics, cash aid to refugees created positive income spillovers for hosts’ businesses and households. However, these benefits were unequally distributed among the local population—locals with better ex-ante access to resources, education, and political connections were more likely to benefit from the arrival of refugees, while the disadvantaged became increasingly vulnerable [9]. Robust analyses of the different dimensions of the impact of refugees on their host population will yield a better picture of how refugees affect their host communities. Such will also show areas where improvements are needed and inform policy and intervention strategies to improve outcomes for both groups.

Importantly, analyses of this nature, assessing the effects of an exposure (such as hosting refugees, as well as other shocks such as droughts, floods, epidemics, etc.) on a community, may shed light on effective methods for empirically measuring resilience as an outcome indicator. In recent years, resilience in food security has been a topic of academic debate and policy interest, albeit with little resolution [10, 11]. One acceptable definition of resilience is the capacity over time of a person, household, or other aggregate units to avoid poverty in the face of various stressors and the wake of a myriad of shocks. Experts consider an entity resilient if that capacity is maintained high over time. This definition captures the notion of resilience in food security [4]. We broaden this view to encapsulate the more encompassing concept of nutrition security, where nutrition security implies food security, and food security, in turn, means nutrient security [12].

Measuring the components of resilience may require different analytic approaches [11]. For nutrition outcomes, the goal remains to trace the ability of communities, households, or individuals to maintain (or improve) baseline nutrition levels over time in the face of stressors and shocks. A difference-in-differences approach provides a logical, analytical framework for using repeated cross-sectional observational data to isolate the effects of exposures over time [13]. When combined with proper matching techniques to create counterfactuals, results from such analyses can approximate those of a quasi-experiment [13]. This approach can offer a way forward when the question is if communities hosting refugees can maintain baseline levels of nutrition outcomes over time, even if suboptimal, for endpoint variables contributing to nutrition security. A necessary condition for this approach is the availability of unexposed communities, households or individuals comparable in important ways to the exposed groups.

In this study, we investigated the impact of refugees on the resilience in nutrition outcomes of a refugee-hosting community in Cameroon. Specifically, we explored effects on (i) maternal underweight, (ii) maternal anaemia, (iii) child wasting; (iv) child stunting; (v) child underweight; and (vi) child anaemia.

## 2 Materials and methods

### 2.1 Study design

We undertook a secondary data analysis using repeated cross-sectional Demography and Health Surveys (DHS) data (data collected from a new sample of participants at successive time points). We also used data on refugee movement, extreme climatic events, epidemics, and conflicts from multiple sources to examine if residents of a refugee-hosting community in Cameroon experienced changes in nutrition status attributable to refugees.

Between 2004 and 2011, Cameroon experienced a rapid inflow of refugees fleeing conflict from the neighbouring Central African Republic. These refugees settled mainly in a geographically circumscribed area in the Eastern region of the country. Using households in the refugee hosting community as the exposure group, we matched households with similar characteristics from the rest of the country as the control group. We used genetic matching algorithms embedded in the Matching R package to select controls with the optimal balance of covariates. Although Cameroon is diverse in terms of culture and climate, we considered our control group appropriate because the central health and development policies apply equally over the national territory. We further undertook an extensive systematic analysis to identify and exclude potential control areas that experienced other shocks, such as political unrest, epidemics, droughts, and floods in the country over the same period (see Supplementary Table S1). We established an exposure and a control group for each study year (2004 and 2011), with 2004 representing the period immediately before the mass migration of refugees and 2011 representing the time after. We conducted a difference-in-differences analysis on nutrition outcomes between the years to estimate the impact of refugees on nutrition outcomes in the refugee-hosting community. We reported this study following the STROBE (STrengthening the Reporting of OBservational studies in Epidemiology) guidelines [14].

### 2.2 Setting

**Figure 1:**
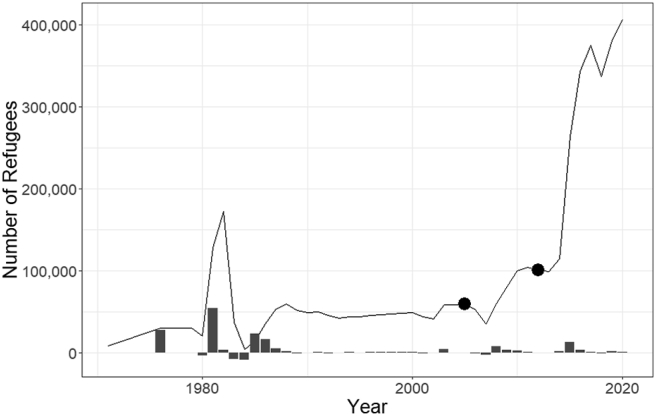
The trend of refugees in Cameroon (data source: www.macrotrends.net).

**Figure 2:**
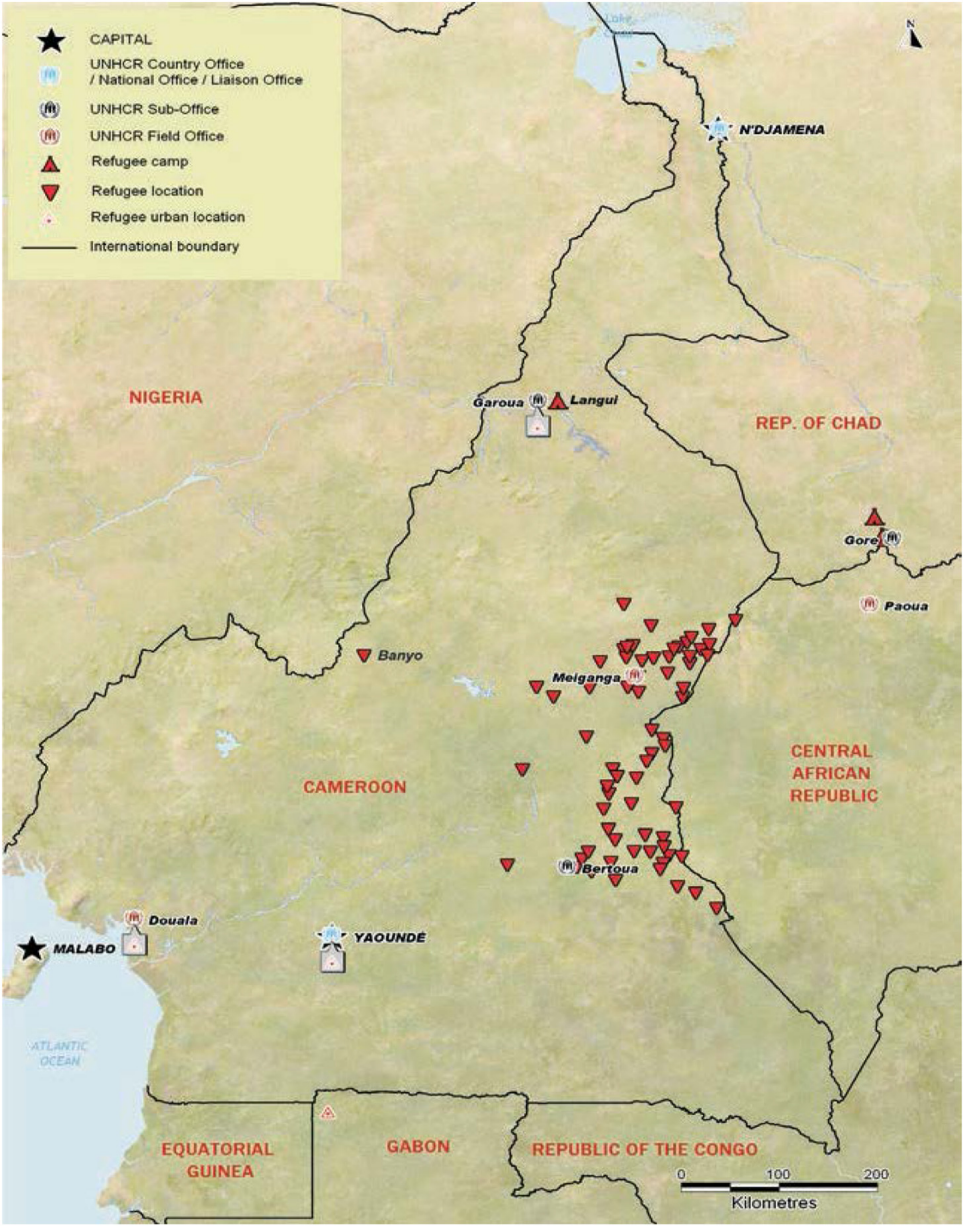
Refugee settlement in Cameroon (extracted from the United Nations High Commissioner for Refugees report, 2011).

Cameroon is a lower-middle-income country in Central Africa, often described as Africa in miniature because of its diverse climate, geography, culture, and politics. The country experiences various shocks, such as intermittent floods, droughts, and epidemic outbreaks, which increase vulnerability to nutrition insecurity, compounding the effects of the low-income status. Until recently, Cameroon has been a peaceful country and has served as a haven for refugees fleeing threats and violence from troubled neighbouring countries. Until 2004, 57000 refugees had settled in three towns in Cameroon (Banyo, Yaoundé and Douala) [15]; the number of settled refugees was relatively stable for a long time before 2004. Nine thousand rural refugees (mainly in Banyo) were from Nigeria. Most of the 49000 urban refugees (mainly in Douala and Yaoundé) were from Chad, the Central African Republic, DRC, Liberia, Rwanda, and Sierra Leone. Since 2006, refugees fleeing the escalating conflict in the Central African Republic have settled in communities on the eastern borders of Cameroon. By 2011, the country’s total number of refugees doubled the 2004 estimate (Figure 1) [16]. The refugee settlement area was largely rural, with few small towns. The small cities rapidly grew as the refugees settled and economic activities increased. While some refugees lived in camps, most lived within the community; they had access to over 2000 ha of arable land offered by the government and local authorities. Support for the refugees was mainly from the Cameroonian government, the United Nations High Commissioner for Refugees (UNHCR) and other NGOs [17]. Based on the 2011 UNHCR report [16], most refugees settled in a well-defined geographical area; their interaction seems to have been with a distinct group. Therefore, we can estimate the impact of their interaction with the host community. Figure 2 shows the UNHCR geolocation of refugee settlement sites in Cameroon in 2011.

### 2.3 Data

Our primary data came from Cameroon’s Demographic and Health Surveys (DHS) of 2004 and 2011 [18]. The DHS is a quinquennial national household survey in over 90 countries sponsored by USAID; it is representative at different subnational levels, thus allowing for varying levels of within-country subgroup analyses. The surveys employ rigorous standardised methodologies, with only minor contextual adaptation, and indicators are comparable across multiple levels. The indicators captured include alcohol consumption, anaemia levels, anthropometry, blood pressure, domestic violence, HIV behaviour, HIV knowledge, HIV status, malaria status, maternal mortality, and tobacco use. Data are accessible on request from the DHS programme. In addition to the DHS data, we used data from the Emergency Events Database (EM-DAT), which maintains an accessible record of emergency events in countries [19], as well as published reports, grey literature and expert knowledge of the context to identify other shocks that occurred in Cameroon around 2004 and between 2004 and 2011.

### 2.4 Participants

Participants analysed in this study were mothers (15–49 years) and children (5 years old and below) who had participated in the 2004 or 2011 DHS. Participants in the 2011 survey were not the same as in 2004 since the DHS uses a repeated cross-sectional design. We focused on mothers and their children because they are more vulnerable to undernutrition during shocks that lead to food and nutrition insecurity. The consequence of undernutrition is most severe in these groups. Refugees were not part of the DHS dataset due to not being Cameroonian nationals.

### 2.5 Patient and public involvement

We analysed data from secondary sources and did not initially engage with the public during the study design and planning phases. The output from this analysis will be available to the population of Cameroon, especially the refugee-hosting communities, through the Ministry of Public Health. In addition, publications from the Demographic and Health Surveys are available on the DHS website for dissemination to the surveyed and broader communities.

### 2.6 Sample size and power calculation

Because this analysis was an ex-post evaluation, the initial sample size constrained the number in the exposed group. We included all individuals surveyed in the refugee area. We increased the total sample size by performing one-exposed to two-control matches. With an average of 800 individuals in the exposed group for each outcome variable, we estimated the average sample of 2400 individuals per outcome. We used a simplistic formula suggested by Hu and Hoover [20] to determine that the sample size had up to 80% power to detect effect sizes as small as 5%.

### 2.7 Outcome variables

The outcome variables for children were weight-for-height (for wasting, and defined as wasted or not), height-for-age (for stunting, and defined as stunted or not), weight-for-age (for underweight, and defined as underweight or not) [21] and anaemia status (defined as anaemic or not). Because of their clinical and public health relevance, we treated all outcome variables as binary. We considered measurements of weight-for-height, height-for-age or weight-for-age that were less than -2 standard deviations from the reference medians to be malnourished. For mothers, we used the body mass index – BMI (and defined as underweight or not) and anaemia status (defined as anaemic or not) as outcome variables. In mothers, BMI ≤18.5 was considered underweight. Despite the known limitations of using BMI as an indicator of nutritional status in the adolescent population, owing to their rapid growth during this time [22], we judged that it was still suitable for comparing group outcomes [23, 24]. All participants who had any form of anaemia (mild, moderate, or severe) were considered anaemic.

#### 2.7.1 Exposure variable

Our exposure variable was the place of residence, i.e., in either the refugee-hosting community or elsewhere in the country, with no other identified shock. We delineated the refugee-hosting community based on the UNHCR 2011 report on Cameroon, which described the refugee areas (Figure 2). Participants were assigned to the refugee hosting community using the quantum geographical information system (QGIS) version 2.14. First, we redistributed sample clusters in the datasets over the national territory using cluster GPS coordinates. Then we considered as exposed all clusters falling within the area mapped out as the refugee area in the UNHCR 2011 country report [16]. Similarly, control participants were selected by matching after mapping and subsequently excluding areas that experienced shocks during the time of interest [25].

#### 2.7.2 Covariates

Our covariates for matching—household characteristics that could predict the outcome—initially included the following: mother’s educational level (no education, primary, secondary, or tertiary education), residence (urban or rural), wealth index (poorest, poorer, middle, richer, or richest), household size, sex of household head (male or female), age of household head, use of bed nets in the household, aridity index and proximity to a water source. These covariates were selected based on literature and a checklist for selecting covariates for difference-in-differences analysis [26, 27]. All selected covariates were initially included for matching, and we subsequently deleted variables that did not help in achieving a balance between the groups in a stepwise manner. The deleted variables were likely to generate a difference in our study groups, but they correlated with other variables. For example, a covariate such as wealth index is from several other potential covariates (including the household’s ownership of selected assets, such as televisions and bicycles; materials used for housing construction; and types of water access and sanitation facilities). These could easily correlate with other variables like household size and the sex of the household head. The final set of covariates ensured that the exposure and control groups had similar background characteristics at baseline and follow-up. These were wealth index, rural/urban residence, household size, mother education, sex of household head, age of household head, aridity index and proximity to a water source.

### 2.8 Statistical analysis

We extracted relevant variables for children and mothers from the respective files of the 2004 and 2011 DHS datasets. We dichotomised outcome variables using public health-relevant cut-off values while maintaining covariates in their original forms. Our exploratory analysis started by evaluating distributions for each numeric variable and frequencies and percentages for each categorical variable. Specifically, we examined numeric variables for normality in distributions and categorical variables for near-zero variance (very few observations in any class) [28]. We explored missing data using a combination of graphical displays and univariate, bivariate and multivariate methods.

We used the GenMatch function in the Matching package in the R statistical software to match controls to exposure [29]. This function uses a genetic search algorithm to determine the weight of each covariate and finds optimal balance using multivariate matching. The genetic algorithm thus allowed for optimised one-to-two nearest neighbour matching. We evaluated the balance of covariates between groups for each outcome using a combination of plots and statistical tests (t-test and Kolmogorov-Smirnov (KS) bootstrap). Once we found balanced controls, we calculated the difference in the proportion of each outcome between groups using the generalised linear model. We calculated the difference-in-differences of outcomes between 2004 and 2011 in per cent point difference. This was the coefficient of the model’s interaction term between year and exposure (exposure∗year). We used a significance level of p-value less than 0.05 and a 95% confidence interval to interpret statistical tests.

For sensitivity analyses, we performed similar calculations in the following instances (i) controls sampled randomly from the rest of the country without matching. Here, we aimed to create comparable groups to evaluate the added value of our matching (ii) by removing the regions with the highest number of disasters from the control pool and (iii) by a stepwise reduction in the number of covariates in the matching.

### 2.9 Research ethics approval

Ethics did not apply to this study since this was a secondary data analysis of anonymised datasets. The DHS programme only provided anonymised datasets on individuals and households. To ensure that we further protected potentially identifiable personal information, we respected the following guidelines during analyses (i) adhered to the DHS data use policy, including sharing data only among registered co-authors and (ii) made no attempt to identify any participant in the anonymised datasets.

## 3 Results

### 3.1 Characteristics of the study population

Table 1 summarises the characteristics of the study population before (2004) and after (2011) the mass arrival of refugees. In 2004, 10656 women and 8125 children under five years were surveyed. Of those, 826 (8%) women and 727 (9%) children resided in refugee-hosting communities. In 2011, 15426 women and 11732 children under the age of five years were studied. Of those, 902 (6%) women and 813 (7%) children resided in refugee-hosting communities. Apart from anaemia which showed a decreasing trend, all the other outcome indicators (wasting, underweight and stunting) showed an increasing trend in the refugee hosting community but a decreasing trend in the rest of the country.

**Table 1:** Characteristics of the populations at baseline (2004) and after refugee arrival (2011), Cameroon Demography and Health Surveys.

	Pre-period (2004)			Post-period (2011)		
	Rest of Country	Refugee Area	p-value	Rest of Country	Refugee Area	p-value
**Women**	9830	826		14524	902	
**Median age in years (IQR)**	25 0 (19 0, 34 0)	26 0 (19 0, 35 0)	0 74	26 0 (20 0, 35 0)	25 0 (20 0, 35 0)	0 102
**Median age household head (IQR)**	40 0 (31 0, 50 0)	39 0 (30 0, 50 0)	0 103	40 0 (33 0, 52 0)	37 0 (30 0, 48 0)	<0 001
**Rural resident (n (%))**	4912 (50 0)	474 (57 4)	<0 001	7164 (49 3)	490 (54 3)	0 004
**Urban type (n (%))**			<0 001			<0 001
Capital city	1935 (19 7)	0 (0 0)		1368 (9 4)	0 (0 0)	
City > 1million	479 (4 9)	0 (0 0)		1460 (10 1)	0 (0 0)	
Small city				1264 (8 7)	0 (0 0)	
Towns	2504 (25 5)	352 (42 6)		3515 (24 2)	412 (45 7)	
Village	4912 (50 0)	474 (57 4)		6917 (47 6)	490 (54 3)	
**Education level (n (%))**			<0 001			<0 001
None	1835 (18 7)	306 (37 0)		2494 (17 2)	302 (33 5)	
Primary	3945 (40 1)	362 (43 8)		5109 (35 2)	371 (41 1)	
Secondary	3841 (39 1)	153 (18 5)		6206 (42 7)	220 (24 4)	
Tertiary	209 (2 1)	5 (0 6)		715 (4 9)	9 (1 0)	
**Household size (median (IQR))**	7 0 (4 0, 9 0)	6 0 (4 0, 10 0)	0 387	7 0 (4 0, 9 0)	6 00 (4 0, 9 0)	0 199
**Under-fives (median (IQR))**	1 0 (0 0, 2 0)	1 0 (0 0, 2 0)	<0 001	1 0 (0 0, 2 0)	1 0 (0 0, 2 0)	0 014
**Female household head (n (%))**	2482 (25 2)	140 (16 9)	<0 001	3912 (26 9)	189 (21 0)	<0 001
**Wealth index (n (%))**			<0 001			<0 001
Poorest	1695 (17 2)	183 (22 2)		2075 (14 3)	217 (24 1)	
Poor	1638 (16 7)	188 (22 8)		2842 (19 6)	211 (23 4)	
Middle	2113 (21 5)	209 (25 3)		2982 (20 5)	206 (22 8)	
Rich	2120 (21 6)	134 (16 2)		3286 (22 6)	157 (17 4)	
Richest	2264 (23 0)	112 (13 6)		3339 (23 0)	111 (12 3)	
**Decision maker on purchases (n (%))**			<0 001			<0 001
Self	1505 (15 3)	129 (15 6)		1484 (16 3)	117 (17 7)	
Husband/Partner	1582 (16 1)	109 (13 2)		3057 (33 6)	214 (32 4)	
Self and Husband	144 (1 5)	2 (0 2)		4306 (47 4)	288 (43 6)	
Someone else	3405 (34 7)	397 (48 1)		202 (2 2)	39 (5 9)	
Other	3053 (31 1)	185 (22 4)		43 (0 5)	2 (0 3)	
Do not know	137 (1 4)	4 (0 5)				
**Smokes (n (%))**	71 (0 7)	5 (0 6)	0 865	33 (0 5)	2 (0 5)	1
**Breastfeeding (n (%))**	2177 (22 1)	216 (26 2)	0 009	3154 (21 7)	256 (28 4)	<0 001
**Have nets in house (n (%))**	2256 (23 0)	196 (23 8)	0 627	4450 (59 4)	240 (51 0)	<0 001
**Under-five under net (mean (SD))**	2 47 (1 01)	2 42 (1 05)	0 215	2 20 (1 17)	2 35 (1 10)	0 001
**Anaemia (n (%))**	2158 (45 6)	160 (40 0)	0 036	2970 (40 4)	158 (33 8)	0 005
**Underweight (n (%))**	265 (5 6)	44 (10 8)	<0 001	441 (5 9)	82 (17 5)	<0 001
**Aridity index (mean (SD))**	47 8 (20 0)	38 7 (4 2)	<0 001	46 34 (21 6)	37 43 (1 8)	<0 001
**Proximity to water (mean (SD))**	95770 (68405 8)	182696 (67160 9)	<0 001	100774 (73173 1)	182991 (61438 8)	<0 001
**Children**	7398	727		10919	813	
**BCG vaccine at birth (n (%))**			<0 001			<0 001
No	1082 (16 3)	135 (20 8)		1268 (12 7)	105 (14 3)	
Yes – recorded and dated	2995 (45 2)	213 (32 9)		4872 (48 7)	292 (39 8)	
Yes – not recorded	2496 (37 6)	289 (44 6)		3819 (38 2)	332 (45 3)	
Yes – not dated	42 (0 6)	10 (1 5)		28 (0 3)	4 (0 5)	
Do not know	15 (0 2)	1 (0 2)		11 (0 1)	0 (0 0)	
**DPT 3 completed (n (%))**			<0 001			<0 001
No	2631 (39 7)	337 (52 1)		3476 (34 9)	262 (35 7)	
Yes – recorded and dated	2434 (36 7)	166 (25 7)		4007 (40 2)	228 (31 1)	
Yes – not recorded	1457 (22 0)	136 (21 0)		2394 (24 0)	239 (32 6)	
Yes – not dated	21 (0 3)	3 (0 5)		22 (0 2)	0 (0 0)	
Do not know	81 (1 2)	5 (0 8)		74 (0 7)	4 (0 5)	
**Has fever (n (%))**			0 403			<0 001
No	4609 (69 7)	467 (72 2)		7109 (71 2)	570 (77 9)	
Yes	1641 (24 8)	150 (23 2)		2546 (25 5)	134 (18 3)	
Do not know	358 (5 4)	30 (4 6)		326 (3 3)	28 (3 8)	
**Stunted (n (%))**	895 (31 2)	102 (33 7)	0 427	1227 (26 3)	126 (34 1)	0 001
**Wasted (n (%))**	142 (4 9)	12 (3 8)	0 478	215 (4 6)	17 (4 6)	1
**Anaemic (n (%))**	2032 (68 1)	214 (69 5)	0 665	2636 (62 2)	199 (60 9)	0 676
**Underweight (n (%))**	475 (16 6)	68 (22 4)	0 013	761 (16 3)	93 (25 1)	<0 001

### 3.2 Shocks in the country during the study period, 2004 - 2011

A detailed review summary of data and reports on shock events that occurred in the country between 2000 and 2011 is included in the supplementary appendix. In brief, we did not identify any shock event in the refugee-hosting community during that time. We identified some important shock events in the Far North, North and Adamaoua regions, but these were difficult to geolocate for exclusion. Ideal control areas free from any reported floods, droughts and epidemics were restricted to the central part of the Adamaoua region and the western part of the East region. Again, these were difficult to carve out geographically. We excluded these regions iteratively from the control to gauge their effects during sensitivity analysis.

### 3.3 Balance in groups after matching

**Figure 3:**
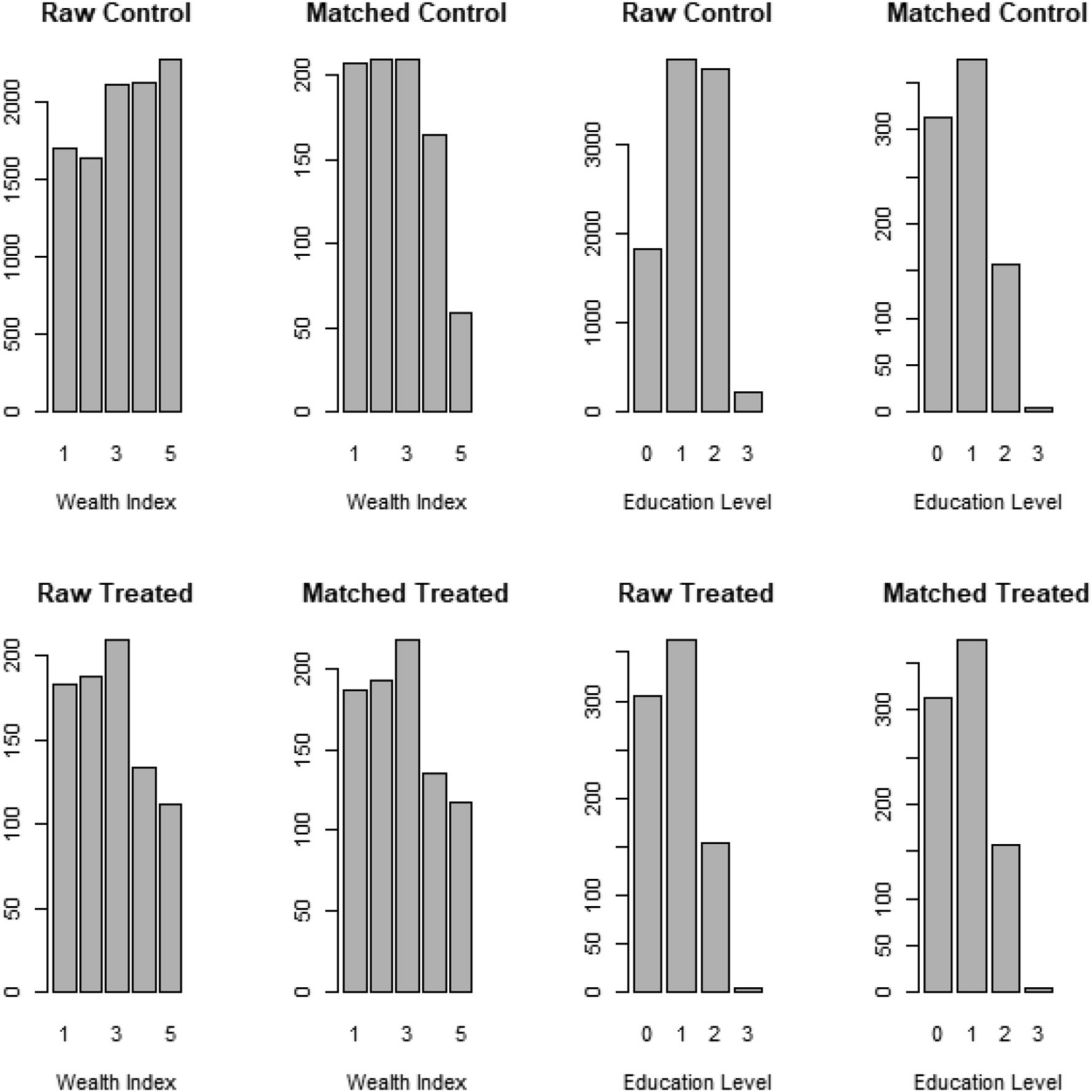
Comparison of wealth index and education level before and after matching between the treated and controls in 2004.

We achieved a good balance in the distribution of the covariates for each indicator in our analyses. Figure 3 shows distributions of two sample covariates for the exposure and control groups before and after an initial 1:1 matching of women’s characteristics in 2004. Before matching, the exposure and control groups differed for four of the eight covariates; after matching, the difference persisted only for two covariates (aridity and proximity to water source). A table of balance for all the used covariates is included in the appendix (supplementary table S4).

#### 3.3.1 Impact of refugees on host’s nutrition

At baseline in 2004 (Table 2), all nutrition outcomes (stunting, underweight, wasting and anaemia in children and anaemia in mothers) were similar for both groups, except underweight in mothers, which was significantly higher in the refugee hosting community.

**Table 2:** Comparison of nutrition outcomes between exposure and matched control at baseline (Cameroon DHS, 2004).

	All regions in control			Few regions∗		
	Control	Exposed	p-value	Control	Exposed	p-value
**Children**	928	928		861	861	
Stunted (%)	92 (28 4)	131 (33 8)	0 145	104 (33 4)	110 (32 1)	0 772
Wasted (%)	15 (4 6)	13 (3 3)	0 474	14 (4 4)	12 (3 4)	0 61
Underweight (%)	66 (20 4)	87 (22 4)	0 567	59 (19 0)	79 (23 0)	0 24
Anaemic (%)	243 (68 8)	266 (69 1)	1	234 (69 2)	249 (70 5)	0 771
**Women**	849	849		820	820	
Underweight (%)	12 (2 6)	46 (11 0)	<0 001	12 (2 8)	48 (12 0)	<0 001
Anaemic (%)	187 (41 1)	162 (39 6)	0 707	184 (42 7)	159 (40 5)	0 563

∗ Adamawa, North and Far North regions were excluded based on higher shock counts (some refugees from Chad and Nigeria, some flood events, and epidemics identified in the review (supplementary appendix S1).

A similar trend of decreasing prevalence of outcome variables was observed in the matched controls. We found no association between changes in the prevalence of maternal underweight, maternal anaemia, child underweight, child anaemia, child wasting, child stunting and the presence of refugees (Table 3).

**Table 3:** Difference-in-differences of nutrition outcomes before and after matching (Cameroon DHS, 2004 and 2011).

	All regions in control				Regions with shock exempted from the control			
	Before Matching		After Matching		Before Matching		After Matching	
	Proportion	p-value	Proportion	p-value	Proportion	p-value	Proportion	p-value
**Stunted children**								
(Intercept)	0 35 (0 30, 0 40)	<0 001	0 28 (0 23, 0 33)	<0 001	0 31 (0 25, 0 36)	<0 001	0 33 (0 28, 0 39)	<0 001
Exposure	-0 01 (-0 09, 0 06)	0 714	0 05 (-0 02, 0 12)	0 126	0 01 (-0 06, 0 09)	0 74	-0 01 (-0 09, 0 06)	0 709
Year	-0 09 (-0 16, -0 01)	0 02	0 02 (-0 05, 0 08)	0 59	-0 11 (-0 18, -0 03)	0 004	-0 04 (-0 10, 0 03)	0 27
exposure:year (DiD)∗	0 09 (-0 01, 0 19)	0 082	-0 02 (-0 10, 0 07)	0 741	0 13 (0 03, 0 23)	0 013	0 08 (-0 02, 0 17)	0 11
**Wasted children**								
(Intercept)	0 02 (0 00, 0 05)	0 03	0 05 (0 03, 0 07)	<0 001	0 05 (0 02, 0 07)	<0 001	0 04 (0 02, 0 07)	<0 001
Exposure	0 01 (-0 02, 0 04)	0 347	-0 01 (-0 04, 0 01)	0 343	-0 01 (-0 04, 0 03)	0 697	-0 01 (-0 04, 0 02)	0 488
Year	0 01 (-0 02, 0 04)	0 375	-0 02 (-0 05, 0 00)	0 112	-0 00 (-0 04, 0 03)	0 788	-0 01 (-0 04, 0 02)	0 594
exposure:year (DiD)	-0 01 (-0 05, 0 04)	0 786	0 03 (-0 01, 0 07)	0 099	0 01 (-0 03, 0 06)	0 659	0 02 (-0 02, 0 06)	0 295
**Underweight children**								
(Intercept)	0 15 (0 10, 0 20)	<0 001	0 20 (0 16, 0 25)	<0 001	0 12 (0 07, 0 16)	<0 001	0 19 (0 15, 0 23)	<0 001
Exposure	0 08 (0 01, 0 14)	0 022	0 02 (-0 04, 0 08)	0 502	0 10 (0 04, 0 16)	0 002	0 04 (-0 02, 0 10)	0 195
Year	0 01 (-0 05, 0 07)	0 714	-0 04 (-0 10, 0 01)	0 134	-0 01 (-0 07, 0 05)	0 642	-0 04 (-0 10, 0 01)	0 135
exposure:year (DiD)	0 02 (-0 07, 0 10)	0 729	0 08 (-0 00, 0 15)	0 058	0 05 (-0 04, 0 13)	0 27	0 06 (-0 01, 0 14)	0 111
**Anaemia children**								
(Intercept)	0 68 (0 63, 0 73)	<0 001	0 69 (0 64, 0 74)	<0 001	0 69 (0 63, 0 74)	<0 001	0 69 (0 64, 0 74)	<0 001
Exposure	0 01 (-0 06, 0 09)	0 718	0 00 (-0 07, 0 07)	0 943	0 01 (-0 07, 0 09)	0 766	0 01 (-0 06, 0 08)	0 715
Year	-0 04 (-0 12, 0 03)	0 25	-0 06 (-0 13, 0 00)	0 064	-0 06 (-0 13, 0 02)	0 131	-0 03 (-0 09, 0 04)	0 441
exposure:year (DiD)	-0 04 (-0 15, 0 06)	0 423	-0 02 (-0 11, 0 08)	0 741	-0 03 (-0 14, 0 08)	0 565	-0 06 (-0 15, 0 03)	0 193
**Underweight women**								
(Intercept)	0 04 (0 01, 0 07)	0 003	0 03 (0 00, 0 05)	0 046	0 05 (0 02, 0 07)	0 001	0 03 (0 00, 0 06)	0 044
Exposure	0 07 (0 03, 0 11)	0 001	0 08 (0 05, 0 12)	<0 001	0 06 (0 03, 0 10)	0 001	0 09 (0 05, 0 13)	<0 001
Year	0 01 (-0 03, 0 05)	0 662	0 02 (-0 01, 0 06)	0 237	-0 02 (-0 06, 0 02)	0 314	0 02 (-0 02, 0 06)	0 243
exposure:year (DiD)	0 06 (0 00, 0 11)	0 037	0 04 (-0 01, 0 10)	0 091	0 08 (0 03, 0 14)	0 002	0 04 (-0 01, 0 09)	0 156
**Anaemia women**								
(Intercept)	0 51 (0 46, 0 56)	<0 001	0 41 (0 37, 0 46)	<0 001	0 51 (0 46, 0 55)	<0 001	0 43 (0 38, 0 47)	<0 001
Exposure	-0 11 (-0 18, -0 04)	0 001	-0 01 (-0 08, 0 05)	0 653	-0 10 (-0 17, -0 03)	0 004	-0 02 (-0 09, 0 04)	0 508
Year	-0 13 (-0 20, -0 07)	<0 001	-0 02 (-0 08, 0 04)	0 578	-0 12 (-0 18, -0 05)	<0 001	-0 07 (-0 13, -0 01)	0 031
exposure:year (DiD)	0 07 (-0 02, 0 16)	0 129	-0 04 (-0 13, 0 05)	0 354	0 05 (-0 04, 0 14)	0 296	0 00 (-0 09, 0 09)	0 996

∗ The interaction term exposure:year (DiD) is the difference-in-difference estimate; i.e., the effect of refugees on the nutrition outcome.

Maternal underweight was 4% (95% Confidence Interval (CI): 1–10%; *P* = 0 091) higher in the refugee hosting community compared to the control group selected from all regions in the country. The difference-in-differences was similar, 4% (CI: -1 – 9%; *P* = 0 156), once we excluded the three regions with the highest number of shocks (Table 3).

On the other hand, maternal anaemia was 4% (CI: -13 – 5%; *P* = 0 354) lower in the refugee hosting community compared to the control group selected from all regions in the country. There was no difference-in-differences, 0% (CI: -9 – 9%; *P* = 0 996), once we excluded the three regions with the highest number of shocks (Table 3).

Child stunting was 2% (CI: -10 – 7%; *P* = 0 741) lower in the refugee hosting community compared to the control group selected from all regions in the country. The difference-in-differences reversed, 8% (CI: -2 – 17%; *P* = 0 11) higher in the refugee hosting community once we excluded the three regions with the highest number of shocks (Table 3).

Child wasting was 3% (CI: -1 – 7%; *P* = 0 099) higher in the refugee hosting community compared to the control group selected from all regions in the country. The difference-in-differences was similar, 2% (CI: -2 – 6%; *P* = 0 295), once we excluded the three regions with the highest number of shocks (Table 3).

Child underweight was 8% (CI: 0–15%; *P* = 0 058) higher in the refugee hosting community compared to the control group selected from all regions in the country. The difference-in-differences was similar, 6% (CI: -1 – 14%; *P* = 0 111), once we excluded the three regions with the highest number of shocks (Table 3).

Child anaemia was 2% (CI: -11 – 8%; *P* = 0 741) lower in the refugee-hosting community compared to the control group selected from all regions in the country. The difference-in-differences increased to 6% (CI: 15–3%; *P* = 0 193) once we excluded the three regions with the highest number of shocks (Table 3).

A further sensitivity analysis (supplementary table S5) with a variable number of covariates showed findings consistent with those reported above. Maternal and child outcome variables did not show substantial differences between the exposure and control groups as we changed our control pool and covariates used for matching.

## 4 Discussion

This study focused on the resilience in maternal and child nutrition outcomes of a refugee-hosting community in Cameroon. It shows overall resilience in maternal and child nutrition outcomes following the arrival of many refugees in the hosting community. When comparing the 2004 and 2011 data, apart from anaemia which showed a decreasing trend, all the other indicators (wasting, underweight and stunting) showed an initial increasing trend in the refugee hosting community but a decreasing trend in the rest of the country. We observed a similar trend in the control group after matching. However, we found no association between changes in the prevalence of maternal underweight, maternal anaemia, child underweight, child anaemia, child wasting, child stunting and the presence of refugees. These findings show resilience in nutrition outcomes as the ability to maintain (or improve) baseline nutrition levels over time in the face of shocks, in this case, the sudden arrival of many refugees in a community. Our approach to excluding areas of concurrent shocks and selecting properly matched controls for our difference-in-differences model offers a way of understanding and estimating resilience.

After excluding the regions exposed to other shocks and matching on selected covariates, the effect sizes for nutrition outcomes were small and pointed in different directions. In children, the prevalence of stunting increased in the refugee hosting community (8%), while the prevalence of anaemia (6%) decreased. In women, there was no change in the prevalence of anaemia but a modest increase in underweight (4%). When comparing these estimates to those obtained from controls selected without excluding areas of increased shock, the variation in point estimates and precisions indicates the results’ importance and sensitivity to concurrent shocks. Isolating a shock event is especially important for estimating resilience in settings where multiple shocks are commonplace.

Comparable studies have observed the impact of refugees on host communities in both directions. Gengo and colleagues (2020) [30] observed improved caloric intake in the Kakuma refugee-hosting community compared to other sites with varying levels of development in Turkana County in Kenya. The Kakuma refugee-hosting community hosts one of the world’s largest refugee camps with 160000 refugees from South Sudan, Somalia, Ethiopia, and the Democratic Republic of Congo [31]. Enhanced nutrition outcomes have been ascribed to better access to cereals through the refugee trade networks and increased employment in the refugee host with the arrival of refugees [31]. On the other hand, food insecurity has been observed in the refugee hosting community in Nimba, Liberia, which hosted refugees from Côte d’Ivoire after the 2010 post-election unrest [32]. Rising food prices with the arrival of refugees are a contributing factor [32]. In the case of nomadic pastoralist hosts, who depend on pasture and water for survival, environmental destruction and soil erosion caused by refugee settlement have been associated with food insecurity [33].

One crucial angle to consider when exploring the impact of refugees on host communities is whether such impacts depend on where the refugees live. Refugees may live in or out of camps, which implies different interactions with the community and, thus different effects. In our case, we had a mix of camped and out-of-camp refugees, with most refugees living out of the camps, as shown in Figure 2. When refugees live in camps, this may imply an intense intervention and less interaction between the refugees and hosts. Conversely, when refugees stay out of camps, there is likely more interaction and exchange of resources. The latter could explain our observed resilience in nutrition outcomes.

The impact of refugees on food and nutrition insecurity is not often spread evenly among the hosts [9], although not explored in the current study, we must acknowledge the importance of this inequity. Gender, age, socioeconomic status, and nature of the host-refugee relationship all affect the spread of benefits and burdens. Hosts with better access to education, resources, and power benefit more, while the disadvantaged are even further marginalised [9]. For example, the increase in food prices in the early stages when refugees arrive may benefit farmers with food surpluses. In contrast, subsistence farmers and landless labourers are often forced to buy at higher prices. Not surprisingly, some relief programs have observed the most vulnerable hosts present for food rations alongside the refugees [34]. A similar phenomenon is observed when abundant local food supplies drive down local food prices, with the familiar disincentive effect of food aid on local food production. The Khartoum refugee conference of 1982 noted that the Ugandan refugees in South Sudan had overgrown cassava to drive down prices in Juba. While this benefited the urban consumers, the local poor Sudanese could not get more from their little surpluses [35].

The implications of these findings are multiple. It is a commonplace for refugee-hosting communities to perceive refugees as a burden, and food is a common area of contention [36]. The persisting nutrition gap between the refugee area and the rest of the country demonstrated in this study represents underlying food insecurity in the refugee host communities, thus a potential source of contention. However, the lack of worsening nutrition security in the refugee host community compared to their matched controls suggests there was no causal link between the arrival of refugees and worsening nutrition status in this population. On the other hand, the lack of significant improvement in the nutritional security of the host community raises questions about the kinds of relief interventions taking place in the area. A proper response to refugees in a well-defined area over a long period should be reflected in the nutrition security of the host, considering the expected changes in the fabric of such a community along the lines of increased economic activities, food supply, and cheap labour [35]. In addition, the communication of the effects of refugees on their host communities needs to acknowledge the absence of a consistent association between the hosting of refugees and poor nutrition outcomes of the host.

Despite filling an important gap in the literature, our study has limitations, most of which are associated with the repeated cross-sectional design and matched controls. First, it used genetic matching to select our controls from the general national population, initially motivated by a large number of individuals in the control group. This method only mimics randomisation by selecting controls with similar chances of residing in the refugee zone based on observed covariates. But other factors that could affect assignment to exposure and outcome that were not observed could not be accounted for in the matching process. Also, any hidden bias due to latent variables could remain after matching despite attempts to include the most comprehensive set of covariates. We still considered this method more appropriate since it has optimal matching strategies compared to alternative designs, such as simple propensity score matching. It improves control of confounding with scarce outcomes and can identify interactions between the propensity of exposure and exposure effect on the outcome [29]. Second, despite our systematic attempt to identify and exclude other areas with relevant shocks during the time of interest, data on such was limited, and controls could still come from internal shocks. In addition, it was challenging to carve out small areas of identified shocks because of limited geographical information. Third, only a few nutrition outcomes were evaluated in this study, limiting any claim of refugee impact on food and nutritional security. However, the indicators examined are sensitive and provide a good sense of nutrition security and resilience. Notwithstanding these limitations, the concepts of humanitarian assistance, the interplay between foreign assistance programs and the host government, and settling refugees in less privileged areas are universal. Further multi-country studies might also be able to assess these contextual factors.

The approach used to estimate nutrition security in the host community raises some further questions that must be addressed. The well-known phenomenon of regression to the mean when matching controls could affect estimates in a difference-in-differences analysis. Although there was no matching of pre-period outcome variables, there was no assessment of any time-varying covariates for low serial correlation, which could also affect estimates [27]. Our power estimation was based on a simplistic suggestion by Hu and Hoover [20], which may overestimate the power compared to the more robust formula proposed by Burlig et al. [37].

## 5 Conclusion

Our findings demonstrate that maternal and child nutrition insecurity in the hosting community does not change as many refugees settle in an area, providing evidence of resilience in these communities. This analysis focused on one refugee site in Cameroon and explored only a few indicators for nutrition security. Analyses, including more indicators in varied settings, are required to assess the robustness of these findings. Nonetheless, our study sheds light on the effect of refugees on communities from a nutrition perspective. It suggests evidence refuting the common perception of refugees as illegitimate interlopers who negatively impact their hosting community. Further studies may benefit from the demonstrated methodology here to conceptualise and assess the resilience of outcomes to exposures of interest (including the effect of policy interventions).

## Declarations

### Author contribution statement

Lambed Tatah, Jose Manuel Rodriguez-Llanes: Conceived and designed the experiments; Performed the experiments; Analyzed and interpreted the data; Contributed reagents, materials, analysis tools or data; Wrote the paper.

Tharcisse Nkunzimana, Alan de Brauw: Conceived and designed the experiments.

Louise Foley: Analyzed and interpreted the data.

### Funding statement

This research did not receive any specific grant from funding agencies in the public, commercial, or not-for-profit sectors.

### Data availability statement

Data associated with this study has been deposited at https://dhsprogram.com/

### Declaration of interest’s statement

The authors declare no conflict of interest.

### Additional information

No additional information is available for this paper.
