# Supplementary material for: Resilience in maternal and child nutrition outcomes in a refugee-hosting community in Cameroon: A quasi-experimental study
Source: Heliyon. 2022 Dec 5;8(12):e12096. doi: 10.1016/j.heliyon.2022.e12096 (PMC9732403; doi:10.1016/j.heliyon.2022.e12096)
Supplement: Supplemental material [file mmc1.docx]

**supplemental material for**

**Resilience in maternal and child nutrition security of a refugee hosting community in Cameroon: a quasi-experimental study**

Lambed Tatah, Tharcisse Nkunzimana, Louise Foley, Alan de Brauw, Jose Manuel Rodriguez-Llanes.

**Corresponding Author Email:** lst36@medschl.cam.ac.uk; tatahlambed@gmail.com.

**Review of climate hazards, epidemics, armed conflicts, and political violence in Cameroon, 2004-2011**

This summary assesses shocks (droughts, floods, and conflicts) in Cameroon during the study period (2004-2011). The aim was to identify areas with significant shocks and exclude them from our analysis based on their scales and ease of delineating them. Considering the prolonged consequences of some shocks, we expanded our search to events occurring from 1997 up to 2011. We used the International EMDAT database to provide an initial overview of shocks in Cameroon. EMDAT lacks detailed spatial information, and that limits its usefulness when it comes to geolocating shock events. The EMDAT search showed that the country was affected by several floods and epidemics (supplementary table 1). Two drought events were reported in 2001 and 2005, but no impact information was provided.

**Supplementary Table S1:** EMDAT Disaster events recorded for Cameroon 1997-2011.

| **Year** | **Disaster type** | **Occurrence** | **Total deaths** | **Total affected** |
| --- | --- | --- | --- | --- |
| 1997 | Epidemic | 2 | 109 | 479 |
| 1998 | Epidemic | 2 | 239 | 2086 |
| 1998 | Insect infestation | 1 | .. | .. |
| 1998 | Transport accident | 1 | 120 | 150 |
| 1999 | Epidemic | 1 | 14 | 105 |
| 1999 | Flood | 1 | 24 | 1000 |
| 1999 | Transport accident | 1 | 29 | .. |
| 1999 | Volcanic activity | 1 | .. | 3010 |
| 2000 | Epidemic | 1 | 22 | 65 |
| 2000 | Flood | 1 | 3 | 500 |
| 2000 | Transport accident | 3 | 51 | 70 |
| 2001 | Drought | 1 | .. | .. |
| 2001 | Epidemic | 2 | 31 | 542 |
| 2001 | Flood | 1 | 30 | 1500 |
| 2003 | Landslide | 1 | 20 | 100 |
| 2003 | Transport accident | 4 | 119 | 44 |
| 2004 | Epidemic | 1 | 46 | 2924 |
| 2005 | Drought | 1 | .. | .. |
| 2005 | Epidemic | 1 | 42 | 1400 |
| 2005 | Flood | 1 | 2 | .. |
| 2005 | Transport accident | 3 | 54 | 12 |
| 2006 | Epidemic | 1 | 8 | 71 |
| 2006 | Transport accident | 4 | 182 | 12 |
| 2007 | Flood | 1 | 8 | 10296 |
| 2007 | Transport accident | 5 | 183 | 55 |
| 2008 | Flood | 1 | 9 | 25000 |
| 2008 | Transport accident | 3 | 67 | 22 |
| 2009 | Epidemic | 1 | 109 | 1456 |
| 2009 | Transport accident | 3 | 36 | 305 |
| 2010 | Epidemic | 1 | 515 | 7869 |
| 2010 | Flood | 1 | 13 | 3095 |
| 2010 | Miscellaneous accident | 1 | .. | 1755 |
| 2010 | Transport accident | 3 | 64 | .. |
| 2011 | Epidemic | 1 | 639 | 16706 |
| 2011 | Transport accident | 3 | 74 | 30 |

Source: EM-DAT: The Emergency Events Database - Université catholique de Louvain (UCL) - CRED, D. Guha-Sapir - www.emdat.be, Brussels, Belgium. Extracted on January 22, 2020

Next, we explored ReliefWeb using the built-in advanced search tool to identify detailed reports on disaster events in Cameroon from 1997 to 2011 (supplementary table 2). Many events on the EMDAT database could be tracked, and most of the reports had more information about the location of the hazards. Most reports were on cholera epidemics and floods, and the events were concentrated in the country's North and Far North regions. The epidemics occurred over consecutive years, and this could significantly affect population health. No information was obtained on droughts from ReliefWeb.

For floods, we searched the Dartmouth Flood Observatory (DFO) database for additional events and their location. The two reported flood events occurred near the coastline in the Southwest part of the country (supplementary table S3). These were found in the EMDAT database but not in Reliefweb.

**Supplementary Table S2.** ReliefWeb Disaster events recorded for Cameroon 1997-2011.

| Year | Disaster type | Location | Deaths | Affected | Link |
| --- | --- | --- | --- | --- | --- |
| 2003 | Landslide | Wabane district,  Lebialem department,  South West province | 20 | 100 | <https://reliefweb.int/disaster/sl-2003-0358-cmr> |
| 2003 | Flood | Magah village,  Lebialem department,  South West province | 20 | 1000 | <https://reliefweb.int/report/burkina-faso/burkina-faso-cameroon-mali-mauritania-niger-senegal-heavy-rains-and-floods-3> |
| 2004 | Insect infestation (locust) | North Province\|  Far North province | .. | .. | [https://reliefweb.int/map/world/sahel-desert-locust-summary\| https://reliefweb.int/map/chad/current-locust-invasion-sep-2004\|https://reliefweb.int/map/burkina-faso/locust-invasion-patterns-plague-outbreak-oct-2004](https://reliefweb.int/map/world/sahel-desert-locust-summary) |
| 2007 | Flood | Mokolo, Kolofata,  Far North province | .. | 10279 | <https://reliefweb.int/report/cameroon/cameroon-floods-dref-operation-no-mdrcm005-final-report> |
| 2008 | Flood | Garoua, North Province | 6 | 5000  (households) | <https://reliefweb.int/report/cameroon/central-africa-chad-and-cameroon-floods-emergency-appeal-no-mdr62003-final-report> |
| 2008 | Epidemic (measles) | Goulfey, Kolofata, Koza,Mada, Makary,  Maroua urbain, Méri,  Mokolo, Mora and Yagoua, NorthProvince | 17 | 355  (cases) | <https://reliefweb.int/report/cameroon/cameroon-measles-information-bulletin-n-2> |
| 2009 | Epidemic (cholera) | North province  Far North Province | 91 | 702  (cases) | <https://reliefweb.int/disaster/ep-2009-000021-cmr> |
| 2010 | Epidemic (cholera) | North province  Far North Province | 621 | 9893  (cases) | <https://reliefweb.int/disaster/ep-2010-000110-cmr> |
| 2010 | Flood | Pouss, Far North Province | 13 | 600  (households) | <https://reliefweb.int/report/cameroon/cameroon-floods-pouss-far-north-region-dref-operation-n%C2%B0-mdrcm010> |
| 2011 | Epidemic (cholera) | North, Far North, Central, Littoral, West and Southwest | 843 | 23152  (cases) | <https://reliefweb.int/disaster/ep-2011-000034-cmr> |

**Supplementary Table S3:** Flood events in Cameroon (1997-2011, Dartmouth Flood Observatory).

| ID | long | lat | Area | Began | Ended | Validation | Deaths | Displaced | Main Cause | Severity |
| --- | --- | --- | --- | --- | --- | --- | --- | --- | --- | --- |
| 1620 | 9.67812 | 3.95416 | 3781.53 | 8/3/2000 | 8/5/2000 | News | 3 | 500 | Heavy rain | 2 |
| 1741 | 8.98727 | 4.4351 | 1086.73 | 6/28/2001 | 6/29/2001 | News | 22 | 1000 | torrential rain | 1 |

We searched the Global Drought Monitor repository for more information on droughts, as droughts are chronic events (as opposed to the more acute shocks described above) with long-lasting impacts on food and nutrition security. We used the standardised precipitation and evapotranspiration index, SPEI-12, to assess the country's occurrence and the regional extent of droughts. Supplementary figure S3 below shows a time series of drought occurrences from 1997 to 2011 in Cameroon. Taking the last twelve months, we focused on annual droughts estimates, which are known to have a wider societal impact. The reference month chosen for the calculations was January of the following year. Overall, the indices show substantial exposure to droughts by large parts of the country during the study period. Additionally, we observed a large variation in exposure across regions that preclude straightforward comparison of individuals sampled from all over the country.

Finally, we assessed the two widely used databases for monitoring conflicts and political violence (Armed Conflict Location and Event Data Project (ACLED) and Uppsala Conflict Data Program (UCDP)) for recorded events in Cameroon. We created buffers of approximately 50km radius to estimate the areas within Cameroon exposed to conflict events which may impact population health. Wagner and colleagues (35) had shown that conflict might have long-lasting impacts (for around seven years) and be far from the violent location (up to 100 km). Supplementary figure S2 shows conflicts and political violence events that occurred in the Northern part of Cameroon from 1997 to 2011. Most of Cameroon was calm during these years compared to its neighbours.

In conclusion, the areas hosting refugees were generally exempted from most other shocks (i.e., floods and epidemics), except for some droughts and a few conflict events. Some areas out of the refugee zone experienced small-scale shocks that were difficult to isolate geographically. We excluded these areas to avoid further confounding by flood, drought, and epidemic exposure. Therefore the control pool excluded Adamaoua, Far North, and Northern regions, despite their similarity to the East region where the refugees resided.


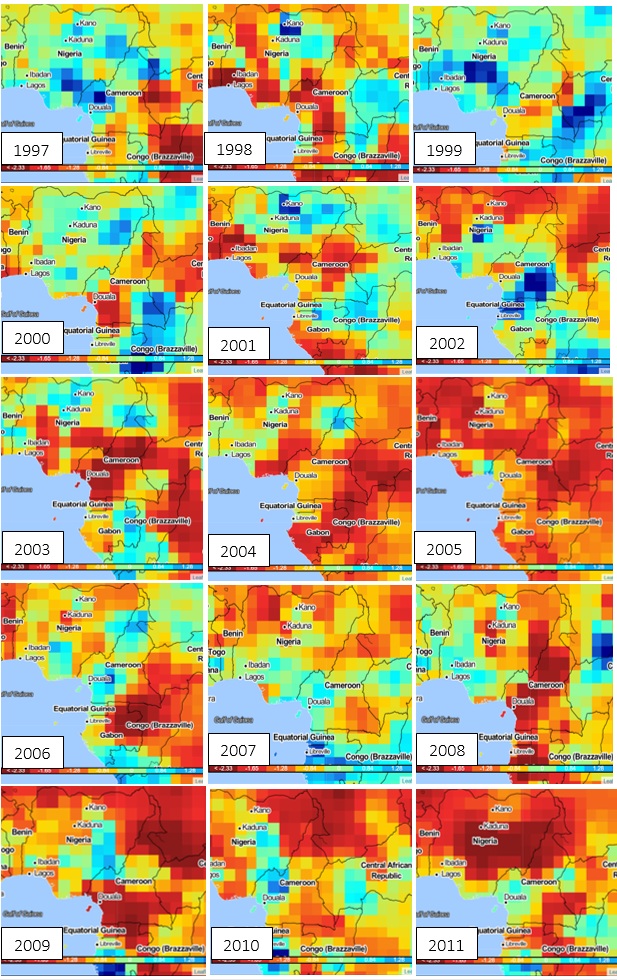


**Supplementary Figure S1:** Droughts in Cameroon from 1997 to 2011 (SPEI-12)


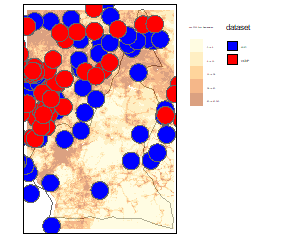


**Supplementary Figure S2.** Violent events in Cameroon, 1997-2011 (UCDP and ACLED)

**Supplementary Table S4.** Match balance for all Covariates and regions

|  | **Before Matching** | **After Matching** |
| --- | --- | --- |
| **Household_head_sex** |  |  |
| Mean exposure | 1·1695 | 1·1695 |
| Mean control | 1·2525 | 1·1598 |
| Std mean diff | -22·109 | 2·5799 |
| Mean raw eqq diff | 0·083535 | 0·0094229 |
| Med raw eqq diff | 0 | 0 |
| Max raw eqq diff | 1 | 1 |
| Mean ecdf diff | 0·0415 | 0·0047114 |
| Med ecdf diff | 0·0415 | 0·0047114 |
| Max ecdf diff | 0·083001 | 0·0094229 |
| Var ratio (Tr/Co) | 0·74664 | 1·0484 |
| T-test p-value | 2·37E-09 | 0·032322 |
| **Age** |  |  |
| Mean exposure | 27·653 | 27·653 |
| Mean control | 27·485 | 27·696 |
| Std mean diff | 1·7459 | -0·45319 |
| Mean raw eqq diff | 0·26877 | 0·40754 |
| Med raw eqq diff | 0 | 0 |
| Max raw eqq diff | 1 | 2 |
| Mean ecdf diff | 0·0078622 | 0·011644 |
| Med ecdf diff | 0·0060419 | 0·0094229 |
| Max ecdf diff | 0·031432 | 0·048292 |
| Var ratio (Tr/Co) | 1·0338 | 1·0863 |
| T-test p-value | 0·62952 | 0·83391 |
| KS Bootstrap p-value | 0·282 | 0·183 |
| KS Naive p-value | 0·43891 | 0·27545 |
| Ks statistic | 0·031432 | 0·048292 |
| **Education_level** |  |  |
| Mean exposure | 0·82688 | 0·82688 |
| Mean control | 1·2466 | 0·82567 |
| Std mean diff | -56·564 | 0·16316 |
| Mean raw eqq diff | 0·41889 | 0·0011779 |
| Med raw eqq diff | 0 | 0 |
| Max raw eqq diff | 1 | 1 |
| Mean ecdf diff | 0·10493 | 0·00029446 |
| Med ecdf diff | 0·099497 | 0 |
| Max ecdf diff | 0·22072 | 0·0011779 |
| Var ratio (Tr/Co) | 0·91505 | 1·0074 |
| T-test p-value | < 2·22e-16 | 0·31731 |
| KS Bootstrap p-value | < 2·22e-16 | 0·999 |
| KS Naive p-value | < 2·22e-16 | 1 |
| Ks statistic | 0·22072 | 0·0011779 |
| **Household_size** |  |  |
| Mean exposure | 7·7337 | 7·7337 |
| Mean control | 7·5145 | 7·4673 |
| Std mean diff | 3·8215 | 4·6453 |
| Mean raw eqq diff | 0·51332 | 0·48292 |
| Med raw eqq diff | 0 | 0 |
| Max raw eqq diff | 19 | 8 |
| Mean ecdf diff | 0·013371 | 0·015269 |
| Med ecdf diff | 0·0087487 | 0·012956 |
| Max ecdf diff | 0·044621 | 0·054181 |
| Var ratio (Tr/Co) | 1·5001 | 1·3053 |
| T-test p-value | 0·28545 | 5·8399E-07 |
| KS Bootstrap p-value | 0·039 | 0·084 |
| KS Naive p-value | 0·096219 | 0·16534 |
| Ks statistic | 0·044621 | 0·054181 |
| **Wealth_index_level** |  |  |
| Mean exposure | 2·7627 | 2·7627 |
| Mean control | 3·1648 | 2·6029 |
| Std mean diff | -30·272 | 12·031 |
| Mean raw eqq diff | 0·40073 | 0·16726 |
| Med raw eqq diff | 0 | 0 |
| Max raw eqq diff | 1 | 1 |
| Mean ecdf diff | 0·080418 | 0·033451 |
| Med ecdf diff | 0·094722 | 0·03298 |
| Max ecdf diff | 0·14816 | 0·068316 |
| Var ratio (Tr/Co) | 0·89722 | 1·1526 |
| T-test p-value | 4·44E-16 | 0·00005861 |
| KS Bootstrap p-value | < 2·22e-16 | 0·006 |
| KS Naive p-value | 5·88E-15 | 0·038038 |
| Ks statistic | 0·14816 | 0·068316 |
| **Urban_rural** |  |  |
| Mean exposure | 1·5738 | 1·5738 |
| Mean control | 1·4997 | 1·5702 |
| Std mean diff | 14·986 | 0·734 |
| Mean raw eqq diff | 0·07385 | 0·0035336 |
| Med raw eqq diff | 0 | 0 |
| Max raw eqq diff | 1 | 1 |
| Mean ecdf diff | 0·037078 | 0·0017668 |
| Med ecdf diff | 0·037078 | 0·0017668 |
| Max ecdf diff | 0·074155 | 0·0035336 |
| Var ratio (Tr/Co) | 0·97927 | 0·99786 |
| T-test p-value | 3·88E-05 | 0·083078 |
| **Aridity** |  |  |
| Mean exposure | 38·661 | 38·661 |
| Mean control | 47·763 | 41·558 |
| Std mean diff | -218·19 | -69·452 |
| Mean raw eqq diff | 16·309 | 5·0487 |
| Med raw eqq diff | 16·529 | 4·2735 |
| Max raw eqq diff | 32·832 | 16·995 |
| Mean ecdf diff | 0·30514 | 0·20853 |
| Med ecdf diff | 0·2442 | 0·16313 |
| Max ecdf diff | 0·73225 | 0·53946 |
| Var ratio (Tr/Co) | 0·043432 | 0·20865 |
| T-test p-value | < 2·22e-16 | < 2·22e-16 |
| KS Bootstrap p-value | < 2·22e-16 | < 2·22e-16 |
| KS Naive p-value | < 2·22e-16 | < 2·22e-16 |
| Ks statistic | 0·73225 | 0·53946 |
| **Proximity_to_Water** |  |  |
| Mean exposure | 182696 | 182696 |
| Mean control | 95770 | 176808 |
| Std mean diff | 129·43 | 8·7676 |
| Mean raw eqq diff | 86932 | 9884·2 |
| Med raw eqq diff | 94365 | 7557·7 |
| Max raw eqq diff | 132121 | 41393 |
| Mean ecdf diff | 0·31247 | 0·045564 |
| Med ecdf diff | 0·33193 | 0·025324 |
| Max ecdf diff | 0·58234 | 0·18021 |
| Var ratio (Tr/Co) | 0·96393 | 1·0037 |
| T-test p-value | < 2·22e-16 | 1·9984E-15 |
| KS Bootstrap p-value | < 2·22e-16 | < 2·22e-16 |
| KS Naive p-value | < 2·22e-16 | 2·1206E-12 |
| Ks statistic | 0·58234 | 0·18021 |

**Supplementary Table S5:** Difference-in-differences before matching and after matching with reduced covariates.

|  | **Before Matching** | | **After Matching (excluding aridity and proximity to water)** | | **Before Matching** | | **After Matching (including only education, wealth, and age)** | |
| --- | --- | --- | --- | --- | --- | --- | --- | --- |
|  | **Percent point difference** | **p-value** | **Percent point difference** | **p-value** | **Percent point difference** | **p-value** | **Percent point difference** | **p-value** |
| **Stunted children** |  |  |  |  |  |  |  |  |
| (Intercept) | 0·33 (0·28, 0·39) | <0·001 | 0·29 (0·24, 0·35) | <0·001 | 0·31 (0·26, 0·37) | <0·001 | 0·31 (0·30, 0·33) | <0·001 |
| exposure | 0·00 (-0·07, 0·08) | 0·956 | 0·04 (-0·03, 0·12) | 0·258 | 0·02 (-0·05, 0·10) | 0·539 | 0·02 (-0·03, 0·08) | 0·429 |
| year | -0·06 (-0·13, 0·01) | 0·112 | 0·00 (-0·07, 0·07) | 0·991 | -0·06 (-0·13, 0·01) | 0·12 | -0·05 (-0·07, -0·03) | <0·001 |
| exposure:year | 0·06 (-0·04, 0·17) | 0·224 | 0·00 (-0·10, 0·10) | 0·946 | 0·06 (-0·04, 0·16) | 0·235 | 0·05 (-0·02, 0·12) | 0·148 |
| **Wasted children** |  |  |  |  |  |  |  |  |
| (Intercept) | 0·06 (0·03, 0·08) | <0·001 | 0·05 (0·02, 0·07) | <0·001 | 0·04 (0·02, 0·07) | 0·001 | 0·05 (0·04, 0·06) | <0·001 |
| exposure | -0·02 (-0·05, 0·01) | 0·281 | -0·01 (-0·04, 0·03) | 0·622 | -0·00 (-0·04, 0·03) | 0·821 | -0·01 (-0·03, 0·01) | 0·413 |
| year | -0·02 (-0·05, 0·01) | 0·244 | 0·01 (-0·02, 0·04) | 0·538 | 0·03 (-0·00, 0·07) | 0·05 | -0·00 (-0·01, 0·01) | 0·616 |
| exposure:year | 0·03 (-0·02, 0·07) | 0·24 | -0·00 (-0·05, 0·04) | 0·908 | -0·03 (-0·07, 0·02) | 0·277 | 0·01 (-0·02, 0·04) | 0·551 |
| **Underweight children** |  |  |  |  |  |  |  |  |
| (Intercept) | 0·17 (0·12, 0·21) | <0·001 | 0·18 (0·14, 0·23) | <0·001 | 0·18 (0·13, 0·22) | <0·001 | 0·17 (0·15, 0·18) | <0·001 |
| exposure | 0·06 (-0·01, 0·12) | 0·088 | 0·04 (-0·03, 0·11) | 0·229 | 0·05 (-0·02, 0·11) | 0·163 | 0·06 (0·01, 0·10) | 0·012 |
| year | 0·01 (-0·05, 0·07) | 0·749 | 0·03 (-0·04, 0·09) | 0·377 | 0·01 (-0·06, 0·07) | 0·813 | -0·00 (-0·02, 0·01) | 0·747 |
| exposure:year | 0·02 (-0·07, 0·10) | 0·714 | -0·00 (-0·09, 0·09) | 0·965 | 0·02 (-0·07, 0·11) | 0·669 | 0·03 (-0·03, 0·09) | 0·328 |
| **Anaemia children** |  |  |  |  |  |  |  |  |
| (Intercept) | 0·67 (0·62, 0·72) | <0·001 | 0·73 (0·68, 0·79) | <0·001 | 0·71 (0·66, 0·77) | <0·001 | 0·68 (0·66, 0·70) | <0·001 |
| exposure | 0·02 (-0·05, 0·10) | 0·525 | -0·04 (-0·11, 0·04) | 0·309 | -0·02 (-0·09, 0·05) | 0·599 | 0·01 (-0·04, 0·07) | 0·601 |
| year | 0·00 (-0·07, 0·08) | 0·937 | -0·08 (-0·15, -0·01) | 0·032 | -0·09 (-0·17, -0·02) | 0·013 | -0·06 (-0·08, -0·03) | <0·001 |
| exposure:year | -0·09 (-0·20, 0·02) | 0·099 | -0·01 (-0·11, 0·10) | 0·91 | 0·01 (-0·10, 0·11) | 0·895 | -0·03 (-0·11, 0·05) | 0·465 |
| **Underweight women** |  |  |  |  |  |  |  |  |
| (Intercept) | 0·05 (0·02, 0·08) | 0·001 | 0·07 (0·06, 0·09) | <0·001 | 0·05 (0·02, 0·08) | 0·001 | 0·06 (0·05, 0·06) | <0·001 |
| exposure | 0·06 (0·02, 0·10) | 0·003 | 0·03 (0·00, 0·07) | 0·044 | 0·06 (0·01, 0·10) | 0·008 | 0·05 (0·03, 0·08) | <0·001 |
| year | -0·01 (-0·05, 0·03) | 0·643 | 0·02 (-0·01, 0·05) | 0·242 | 0·01 (-0·03, 0·05) | 0·626 | 0·00 (-0·00, 0·01) | 0·359 |
| exposure:year | 0·08 (0·02, 0·13) | 0·006 | 0·05 (-0·00, 0·10) | 0·05 | 0·06 (0·00, 0·11) | 0·046 | 0·06 (0·03, 0·10) | <0·001 |
| **Anaemia women** |  |  |  |  |  |  |  |  |
| (Intercept) | 0·42 (0·38, 0·47) | <0·001 | 0·46 (0·43, 0·49) | <0·001 | 0·42 (0·38, 0·47) | <0·001 | 0·46 (0·44, 0·47) | <0·001 |
| exposure | -0·02 (-0·09, 0·04) | 0·478 | -0·06 (-0·11, -0·00) | 0·034 | -0·02 (-0·09, 0·04) | 0·488 | -0·06 (-0·11, -0·01) | 0·029 |
| year | -0·05 (-0·12, 0·01) | 0·111 | -0·04 (-0·09, 0·01) | 0·096 | -0·05 (-0·11, 0·02) | 0·147 | -0·05 (-0·07, -0·03) | <0·001 |
| exposure:year | -0·01 (-0·10, 0·08) | 0·833 | -0·02 (-0·10, 0·06) | 0·627 | -0·01 (-0·11, 0·08) | 0·764 | -0·01 (-0·08, 0·06) | 0·769 |
